# Evaluation of Circulating MicroRNA Biomarkers in the Acute Pancreatic Injury Dog Model

**DOI:** 10.3390/ijms19103048

**Published:** 2018-10-06

**Authors:** Han-Byul Lee, Hyun-Kyu Park, Hyun-Ji Choi, Sora Lee, Sang-Joon Lee, Ji-Young Lee, Eun-Ho Cho, Hyo-Jeong Han, Ju-Hyung Seok, Woo-Chan Son

**Affiliations:** 1 Asan Institute for Life Sciences, Asan Medical Center, Seoul 05505, Korea; onestar0620@naver.com (H.-B.L.); nevok@hanmail.net (H.-K.P.); snapple08@naver.com (H.-J.C.); bosomdasom@gmail.com (S.L.); tkdwns516@gmail.com (S.-J.L.); goodbye9068@hanmail.net (J.-Y.L.); jhonho0724@gmail.com (E.-H.C.); hhyoj91@gmail.com (H.-J.H.); sjh4409@gmail.com (J.-H.S.); 2 Department of Pathology, Asan Medical Institute of Convergence Science and Technology, Asan Medical Center, University of Ulsan College of Medicine, Seoul 05505, Korea

**Keywords:** miRNA-216a, miRNA-375, miRNA-7, miRNA-551b, pancreatitis, beagle dog

## Abstract

This study aimed to evaluate the usefulness of four microRNAs (miRNAs) in an acute pancreatic injury dog model. Acute pancreatitis was induced by infusion of cerulein for 2 h (7.5 μg/kg/h). The levels of well-known miRNAs, microRNA-216a (miR-216a) and microRNA-375 (miR-375), and new candidates microRNA-551b (miR-551b), and microRNA-7 (miR-7), were measured at 0, 0.5, 1, 2, 6, 12, and 24 h with serum amylase and lipase, and histopathological examination was performed. Among the four miRNAs, miR-216a and miR-375, and serum enzymes were significantly increased by cerulein treatment. The expression levels of miRNAs and serum enzymes peaked at 2–6 h with a similar pattern; however, the overall increases in miR-216a and miR-375 levels were much higher than those of the serum enzyme biomarkers. Increased levels of miR-216a and miR-375 were most highly correlated to the degree of individual histopathological injuries of the pancreas, and showed much greater dynamic response than serum enzyme biomarkers. Twenty-four-hour time-course analysis in this study revealed time-dependent changes of miRNA expression levels, from initial increase to decrease by predose level in acute pancreatitis. Our findings demonstrate that, in dogs, miR-216a and miR-375 have the potential to sensitively detect pancreatitis and reflect well the degree of pancreatic injury, whereas miR-551b and miR-7 do not.

## 1. Introduction

Some commonly prescribed medications are associated with drug-induced pancreatic injury, and mortality reaches 30% in patients with severe pancreatitis in serious cases [1,2]. Damage to the exocrine pancreas causes an inappropriate release of digestive enzymes and subsequent injury to the pancreas itself or other organ systems. The classical biomarkers of pancreatitis, amylase and lipase, have some limitations related to sensitivity and specificity [3,4]. Their slow clearance cannot reflect the exact condition of the patient. In addition, increases of these serum enzymes can occur in nonspecific pathologic conditions, such as salivary gland disease, intra-abdominal inflammation, diabetic ketoacidosis, acute liver failure, and chronic renal failure [5,6].

miRNAs are small noncoding or non-messenger RNAs that regulate gene expression by binding to mRNAs and interfering with their translation [7]. Some miRNAs are abundantly expressed in a tissue-specific manner, and resistant to degradation in the serum. Also, it is known that miRNAs are well-conserved across species [8], so miRNAs obtained from a simple blood draw can be useful translational biomarkers for monitoring tissue injury [9]. Previous studies attempted to identify and validate the usefulness of miRNAs as biomarkers of pancreatitis in rats [9,10,11,12,13,14], and humans [15,16,17,18]. The levels of exocrine pancreas-enriched miR-216a, miR-216b, and miR-217 and endocrine pancreas-enriched miR-375 increase following pancreatic damage. Circulating miR-216a was detectable in all patients with acute pancreatitis; however, the ability of this miRNA to differentiate between moderate and severe pancreatic damage was inconsistent [16,17]. In addition to these well-known miRNAs, two new candidate miRNA biomarkers of pancreatic damage have been identified in humans. It has been suggested that miR-551b is associated with severity and miR-7 can be used for early detection of pancreatitis [17,18]. These human studies demonstrated a potential value of miR-216a in the diagnosis of pancreatitis, and the results were consistent with those of studies using animal models of pancreatitis. However, their findings were hampered by insufficiencies such as small sample sizes, lack of correlation with histological changes, and inadequate time points. Therefore, preclinical studies using animal models performed in diverse experimental conditions would facilitate more accurate data analysis for comparison of human and animal miRNA data.

In beagle dogs, which are one of the most frequently used nonrodent animals in preclinical studies, miR-216a and -375 are specifically highly tissue-enriched in the pancreas [19]. Several miRNAs, including miR-216a, -216b, -217, -141, -148a, and -375, have been assessed for their responsiveness to pancreatic injury in dogs [9,20]. Very little is known about miR-551b and -7 in dogs. In this study, we evaluate the fluctuations of four selected miRNAs compared with serum pancreatic injury biomarkers, amylase and lipase, in a beagle dog pancreatitis model. To date, most studies examining miRNA levels in animal models have been performed in rodent models [9,10,11,12,13,14] and only limited data are available in beagle dogs [9,20]. To use miRNAs as biomarkers in preclinical studies, it is important to identify translatability from animal models to humans. The objectives of this study were to examine the levels of two well-known miRNAs, miR-216a and miR-375, and two new candidate miRNAs, miR-551b and miR-7, and evaluate their usefulness as potential biomarkers for acute pancreatic injury in preclinical studies. Furthermore, we compared the levels of these miRNAs with those of serum amylase and lipase, the conventional biomarkers of pancreatic injury, and assessed concordance of the results with histopathological analyses of the pancreas to evaluate the superiority of miRNAs as candidate biomarkers.

## 2. Results

### 2.1. Clinical Signs

Salivation (7/8 dogs) and vomiting (6/8 dogs) were the major clinical signs of acute pancreatitis observed in the cerulein-treated dogs. Vomiting, which was most pronounced in treated animal T8, occurred 0.5–2 h after the start of the infusion, and disappeared thereafter. The animals showing minimal responses were T5 (no clinical signs) and T6 (salivation only). The control animals showed no salivation or vomiting.

### 2.2. Time-Course Changes in Serum Chemistry

The serum levels of alkaline phosphatase (ALP), aspartate transaminase (AST), alanine aminotransferase (ALT), blood urea nitrogen, total cholesterol, creatinine, and glucose were examined at the 2 h time point. Minimal increases in AST and ALT levels were observed in animals T2 and T8. Specifically, their AST levels increased by 2.5-fold and 2.8-fold, respectively, compared with the control group. Similarly, their ALT levels were 2.2-fold and 3.9-fold higher than the mean level in the controls. There were no statistically significant differences between the control and treated animals for the serum chemistry parameters examined.

### 2.3. Time-Course Changes in Serum Enzyme Biomarkers

**Figure 1:**
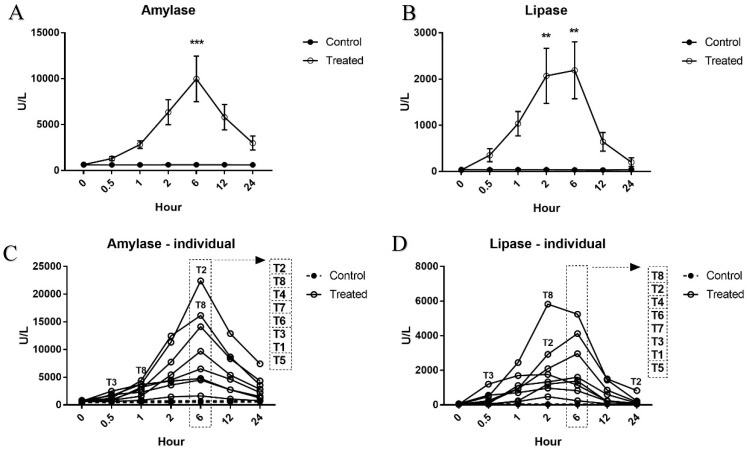
Time-course changes in mean and individual serum amylase and lipase levels. (**A**) amylase; (**B**) lipase; (**C**) amylase–individual; (**D**). lipase–individual. Absolute values (U/L) of serum amylase and lipase levels are shown as mean ± standard error. In each individual graph, the order of response of individual animals at the 6 h time point is shown in a dotted rectangle. ** *p* < 0.01 and *** *p* < 0.001 compared to vehicle controls.

The serum levels of amylase and lipase increased as early as 0.5 h after the start of the cerulein infusion, and peaked at 6 h (Figure 1A, B). At the 6 h time point, the mean serum amylase and lipase levels were 15-fold and 67-fold higher, respectively, than those of the vehicle controls. The levels of these enzymes in the treated animals were statistically significantly higher than those in the vehicle control animals at 6 h for amylase (*p* < 0.001), and 2 h and 6 h for lipase (*p* < 0.05). In addition, the levels of these enzymes significantly increased at 2, 6, and 12 h for amylase (*p* < 0.001 or 0.0001) and 2 h and 6 h for lipase (*p* < 0.0001) compared with those at baseline (0 h). At the 24 h time point, the serum levels of amylase and lipase in the treated animals tended to return to predose values. The time course and magnitude of change in amylase and lipase over time at the individual animal level are depicted in Figure 1C, D. At the 6 h time point, the magnitude of increases ranged from 3-fold to 35-fold for amylase, and 13-fold to 163-fold for lipase. The maximal increase of the two enzymes was noted in T2 and T8, respectively, and T5 was the most minimally affected for both enzymes. The control animals showed relatively minor variations in amylase (430–830 IU) and lipase (20–67 IU) levels at all time points examined.

### 2.4. Time Course of Changes in Serum miR-216a, miR-551b, miR-375, and miR-7 Levels

**Figure 2:**
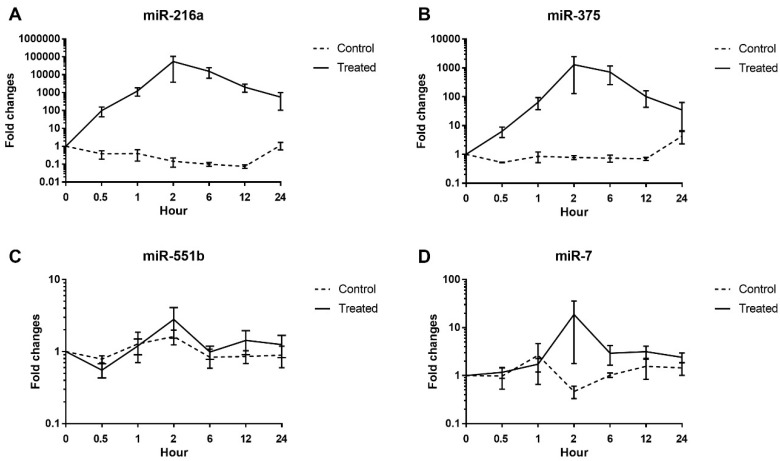
Time-course changes in mean serum miRNAs levels. (**A**) miR-216a; (**B**) miR-551b; (**C**) miR-375; (**D**). miR-7. Fold changes from baseline levels (0 h) were calculated. Data are shown as mean ± standard error.

Circulating miR-216a, miR-551b, miR-375, and miR-7 levels were examined at multiple time points after cerulein or saline infusion of the dogs (Figure 2). Cerulein treatment induced marked increases in miR-216a and miR-375, and a minor increase in the miR-7 level; however, the level of miR-551b was not affected. The maximum mean changes in expression levels were 54,906-fold for miR-216a, 1307-fold for miR-375, and 18-fold for miR-7. The initial increases in miRNA expression levels occurred at the 1 h time point, and the levels tended to decrease close to baseline at 24 h.

**Figure 3:**
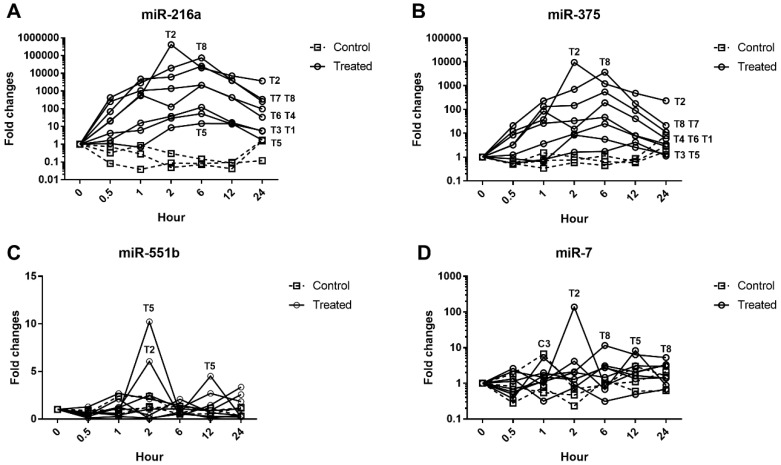
Time-course changes in individual serum miRNAs levels. (**A**) miR-216a; (**B**) miR-551b; (**C**) miR-375; (**D**). miR-7. Fold changes from individual baseline levels (0 h) were calculated. The order of response of individual animals at the 2 h time point is shown in the dotted rectangle.

Increases in the expression levels of miRNAs were also evaluated at the individual animal level (Figure 3). At 2 h, the largest increases were seen in animal T2 (412,023-fold for miR-216a, 9537-fold for miR-375, and 137-fold for miR-7) and the individual magnitudes of fold change for miR-216a and miR-375 were as follows: T8 >> T7 > T6 > T4 > T1 or T3 > T5.

**Figure 4:**
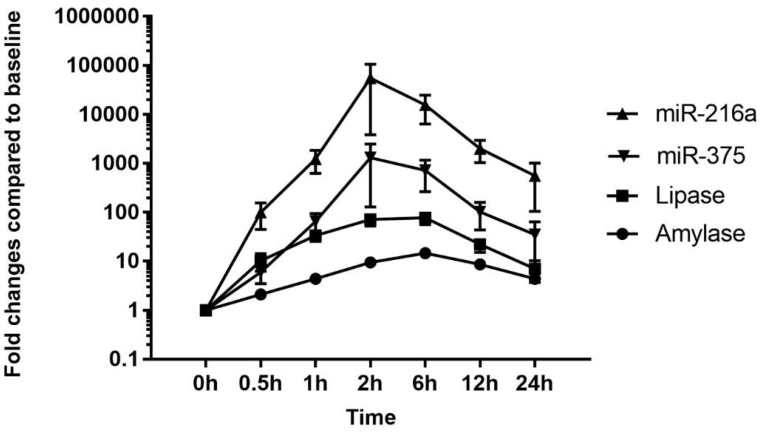
Comparison of miRNA levels and serum chemistry parameters. Mean fold changes in serum miR-216a, miR-375, amylase, and lipase levels. Time course of fold changes compared with baseline levels (0 h).

In all other animals except T2, the expression levels of miR-216a, miR-375, and miR-7 peaked at 6 h, which is the same time point that peak increases occurred in serum amylase and lipase; however, the overall increases in miR-216a and miR-375 levels were much higher than those of the serum enzyme biomarkers (Figure 4). In the control animals, the miRNA responses were more variable than those of serum amylase and lipase, and the largest changes observed were 26-fold for miR-216a, 8-fold for miR-375, and 4-fold for miR-7.

### 2.5. Histopathology of the Pancreas

**Figure 5:**
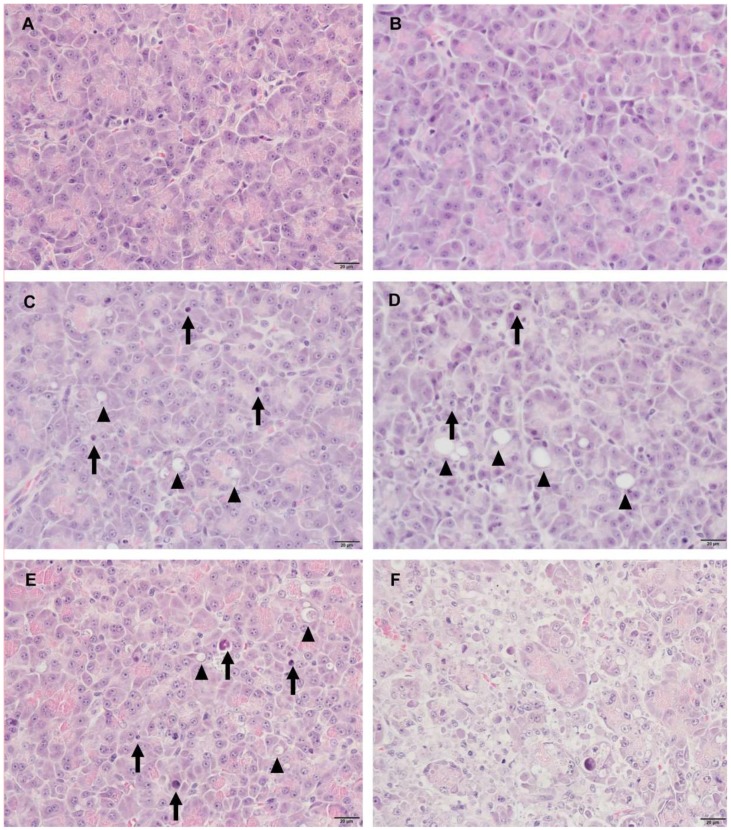
Representative histological images of the pancreas stained with hematoxylin and eosin, 400×. (**A**) Control animal. The acinar cells displayed normal architecture. (**B**) T5 showed only minimal acinar cell degranulation. (**C**) T4, (**D**) T7, and (**E**) T8. Variable slight to moderate effects were characterized by reduced numbers of eosinophilic zymogen granules, pyknotic nuclei with shrunken cytoplasm (arrows), and atrophic and/or vacuolated cytoplasm (arrowheads). (**F**) T2. Massive necrosis with apoptosis of acinar cells occurred in the lobular area and normal architecture was disrupted.

**Figure 6:**
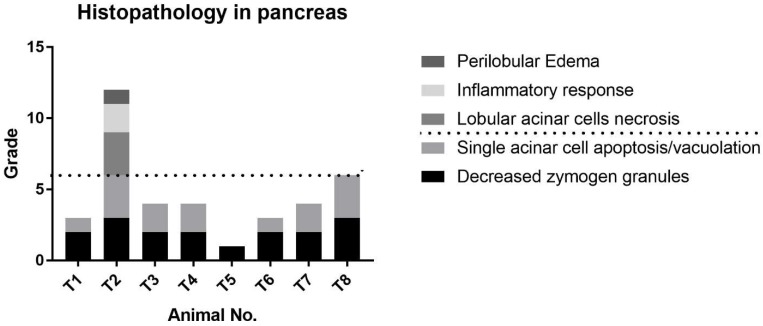
Histopathological analysis of the pancreas. Scores for pancreatic lesions were summed and total score is shown. Dotted line distinguishes T2 from the other treated animals based on the observed perilobular edema, inflammatory response, and lobular acinar cell necrosis.

A variable minimal to moderate degree of decreased eosinophilic zymogen granules and acinar cell apoptosis/vacuolation were observed in most of the cerulein-treated animals, and the findings were characterized by pyknotic nuclei, apoptotic bodies, and/or cytoplasmic vacuolations (Figure 5). These lesions were related to a reduction in the size of the acinar cell, and pancreatic lobules appeared basophilic and atrophic. In the most severely affected animal, T2, there was a notable focal area of necrosis, inflammation, and perilobular edema, which were not seen in the other cerulein-treated animals (Figure 5D and Figure 6). At the individual animal level, the degree of cerulein-induced histopathological effects on the pancreas was T2 >> T8 > T7 > T3 = T4 > T1 = T6 > T5, and was well-correlated with changes of miR-216a and miR-375. No treatment-related lesions were observed in the control animals.

### 2.6. Relative Fold Changes in Serum miRNAs and Enzymes in Severely Affected Animals

**Figure 7:**
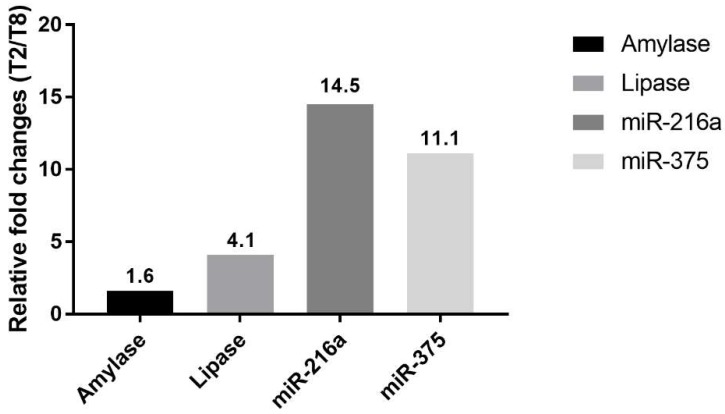
Comparison of miRNA levels and serum chemistry parameters in animals T2 and T8. Relative fold changes at 24 h are shown.

The relative fold changes of miRNAs and serum enzyme biomarkers were additionally analyzed in T2 and T8 to verify the correlation with the histopathological examination results at 24 h. The expression levels of miR-216a and miR-375 in animal T2 at 24 h were 14.5-fold and 11.1-fold higher, respectively, than those in T8, a moderately affected animal (Figure 7). Compared with those of the miRNAs, the relative fold changes of serum amylase and lipase were not remarkable.

## 3. Discussion

To date, most studies of miRNAs have been performed in rodent models, and only a few have been conducted in beagle dogs [9,20], one of the most frequently used nonrodent animals in preclinical studies. Evaluation of miRNA in beagle dogs is important in order to use this biomarker in nonrodent preclinical toxicology studies and to aid translation of the findings to humans. The results presented here confirm that miR-216a and miR-375 are potential candidate biomarkers of pancreatic injury in dogs, whereas miR-551b and miR-7 are not (Figure 3). In addition, when compared alongside histopathological changes of the pancreas for each animal, the miR-216a and miR-375 levels reflected well the acute injury state of the pancreas better than the serum enzyme biomarkers (Figure 5, Figure 6 and Figure 7).

The serum levels of miR-216a, miR-375, amylase, and lipase were increased from 0.5 h after cerulein treatment and tended to decrease. There were some notable findings regarding the changes of the candidate miRNAs and the traditional serum enzyme biomarkers. First, at the individual animal level, the patterns of change in miRNAs were similar to those of serum enzymes, although there was wide interindividual variability. Based on individual responsiveness of miRNAs and conventional serum biomarkers, 8 treated animals could be classified into three response groups: high responders (T2 and T8), moderate responders (T4, T6, and T7), and low responders (T3, T1, and T5) (Figure 1 and Figure 3). This classification is also consistent with histopathology examination, and miRNAs were more highly correlated with pancreatic tissue damage (Figure 5, Figure 6 and Figure 7). Second, the dynamic range of response was wider in miR-216a and miR-375 compared with those of serum amylase and lipase after cerulein infusion (Figure 4). This result is consistent with those of previous studies [10,11,13,20] and indicates high sensitivity of miRNAs as biomarkers of acute pancreatitis. In addition, these dynamic changes of miRNAs indicate the possibility of early detection of acute pancreatic damage.

Cerulein, a cholecystokinin agonist, induces pancreatitis by causing excessive secretion of zymogen granules and subsequent autophagy or apoptosis of acinar cells. Among the treated animals, T2 was the most severely affected, followed by T8; however, there was a substantial difference in histopathological lesions. Including T8, most of the animals presented limited changes: single-cell apoptosis/vacuolation in acinar cells and decreased zymogen granules. In T2, on the other hand, marked necrosis disrupted normal lobular architecture, including acinar cells, islet cells, vasculature, and duct system. Since there were no apparent differences in serum levels of miRNAs and enzymes between T2 and T8, we compared their relative fold changes in biomarkers (Figure 7). The relative changes of miR-216a and miR-375 in T2, which had a severely damaged pancreas, were 14.5-fold and 11.1-fold higher, respectively, than those in animal T8. Considering the massive difference in histological changes of T2 and T8, serum levels of miR-216a and miR-375 were better indicators of the degree of pancreatic injury than the serum enzyme biomarkers. Furthermore, compared with the considerable difference in histopathological tissue damage of T2 and T8, their relative fold changes between miR-216a and 375, 14.5-fold and 11.1-fold, respectively, were not that big. It suggests that, in the case of sensitive miRNA, serum level increasing more than 10 times could reflect drastic tissue changes when some degree of acute pancreatic damage exists.

Recently, Rouse et al. performed a similar analysis of candidate miRNA biomarkers of pancreatic injury in dogs [20]. This study found that the levels of exocrine pancreas-enriched miR-216a, miR-216b, and miR-217, and endocrine pancreas-enriched miR-375 and miR-148a, showed similar or greater sensitivity, a larger range of response, and higher correlation with acinar cell injury than those of serum amylase and lipase. The results in the present study support those findings; however, there are some differences. Rouse et al. induced pancreatitis by administering subcutaneous injection, monitored every 3 h, used droplet digital PCR instrumentation, and examined a different panel of miRNAs. Furthermore, the present study shows substantial correlation between miRNA biomarker levels and histopathological effects at the individual animal level. Our study demonstrates that miR-216a and miR-375 levels are good indicators of the injury state of the pancreas in beagle dogs. Our extended 24 h time-course analysis revealed whole time-dependent changes of miRNA expression levels from initial increase to decrease by predose level.

In humans, miR-216a levels increase in patients with acute pancreatitis [15,16,17]. In a study by Zhang et al. [16], patients were grouped into those with mild acute pancreatitis (MAP), moderately severe acute pancreatitis (MSAP), and severe acute pancreatitis (SAP). miR-216a differentiated initial stage SAP from MAP and MSAP, but could not discriminate MAP and MSAP, which was consistently observed in another study by Blenkiron et al. [15]. The results of these human studies demonstrate the use of miR-216a in the early diagnosis of pancreatitis and are consistent with those of animal models of pancreatitis; however, the human studies had a number of insufficiencies, including small sample size, lack of histology correlation, and the use of inadequate time points. Therefore, supportive animal studies conducted in various experimental conditions will allow more convincing comparisons between species. In addition to miR-216a, human studies have suggested that increased serum levels of miR-551b are associated with severity of pancreatitis [17,18], and decreases in miR-7 [18] can be used to detect the early phase in patients with acute pancreatitis. In the current study, however, the serum level of miR-551b was not significantly affected by cerulein treatment. In the most severely affected animal (T2), the serum level of miR-7, a known regulator of pancreatic β-cell function [21,22], was increased by approximately 100-fold at 2 h, rather than decreased. Very little is known about miR-551b and -7 in dogs at present, and relatively weak fluctuation of these miRNAs was seen in our study. Further study is needed to address these species differences; however, in this study condition, those miRNAs do not seem to be sensitive for evaluating acute pancreatitis in dogs.

In conclusion, our study further supports the notion that miR-216a and miR-375 could be used as circulating serum biomarkers to aid in the detection of acute pancreatitis. These miRNAs may be able to detect the degree of injury more sensitively than traditional biomarkers, and may be useful for monitoring idiosyncratic pancreatitis or the effects of compounds known to target the pancreas, such as incretin-based compounds, during the drug development process. Further validation studies in clinics, or using different animal models and experimental conditions, are required to validate our findings.

## 4. Materials and Methods

### 4.1. Animals

Eleven adult male beagle dogs, aged 1–2 years and weighing 9.5–11.3 kg, were used in this study. The dogs exhibited normal physical examination findings, including body temperature, body weight, and general behavior. Each dog was housed individually in a cage in an air-conditioned room at 23–27 °C with 40–60% humidity. The dogs had access to Canine LabDiet^®^ 5007 (PMI Nutrition International Inc., St. Louis, MO, USA). Water was available ad libitum. All animal experiment protocols were performed in agreement with the guidelines established by the Institutional Animal Care and Use Committee of Orient Bio Inc. (Seongnam-Si, Korea) and were preapproved by the institutional review board (ORIENT-IACUC-16108, 27 May 2016).

### 4.2. Study Design

**Figure 8:**
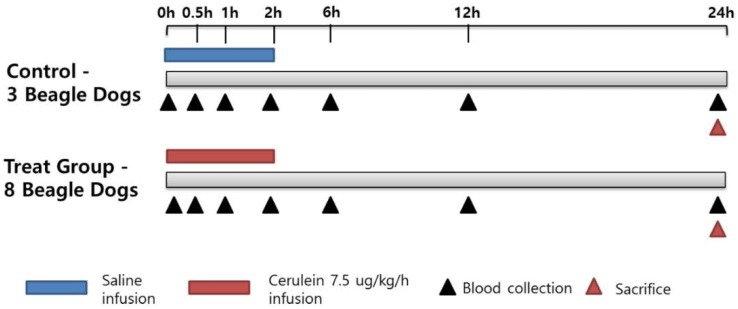
Study design. Eleven beagle dogs (3 control and 8 treated) were intravenously infused with normal saline or cerulein at 7 µg/kg/h for 2 h. Blood was collected at 0, 0.5, 1, 2, 6, 12, and 24 h after the infusion.

The study design (Figure 8) included 3 control and 8 treated animals (C1–C3 and T1–T8, respectively). Acute pancreatitis was induced in the treated group by continuous intravenous infusion of cerulein in saline into the cephalic vein at a rate of 3 mL/kg/h (7.5 μg/kg/h) for 2 h. The dose was selected based on the successful induction of acute pancreatitis in dogs in a previous study [23]. The control animals received a saline infusion under the same conditions. Clinical observations included presence of clinical signs associated with acute pancreatitis, such as vomiting or diarrhea. After the start of the infusion, food was withheld for 24 h, and blood was collected at 0, 0.5, 1, 2, 6, 12, and 24 h. The animals were euthanized and necropsied at the 24 h time point. At necropsy, the pancreas was collected for histopathological examination.

### 4.3. Serum Biomarkers

Serum amylase, lipase, ALP, ALT, AST, blood urea nitrogen, total cholesterol, and creatinine levels were analyzed by Green Cross Lab Cell Corporation (Yongin, Korea) using an automated clinical chemistry analyzer (Modular Analytics, Roche, Germany). Serum amylase and lipase levels were evaluated 0, 0.5, 1, 2, 6, 12, and 24 h after the start of the infusion, and the other parameters were examined at the 0 and 2 h time points.

### 4.4. Serum miRNA Isolation and Reverse Transcription

Small RNA was prepared from 300 μL serum using the NucleoSpin miRNA Plasma Kit (Macherey-Nagel, Düren, Germany; Catalog No. 740971) according to the manufacturer’s protocol for small RNA and DNA purification from plasma or serum (User Manual: February 2016/Rev. 04). cDNA was synthesized from total RNA using a TaqMan^®^ MicroRNA Reverse Transcription Kit (Applied Biosystems, Foster City, CA, USA; Catalog No. 4366596) according to the manufacturer’s instructions (User Manual No. 4364031 Rev. E, 01/2011, pages 13–15). The RNA samples were quantified using a NanoDrop 2000 spectrophotometer (Thermo Fisher Scientific, Waltham, MA, USA). Reverse transcription (RT) was carried out in a total volume of 15 µL. Briefly, 5 µL undiluted total RNA was added to 7 µL master mix, which comprised 3 µL of 5× multiplex RT primer pool, 0.15 µL of 100 mM deoxyribonucleotide triphosphates (dNTPs), 1 µL of 50 U/µL MultiScribe reverse transcriptase, 1.5 µL of 10× RT buffer, 0.19 µL RNase inhibitor, and 4.16 µL RNase-free water. The reaction was mixed, placed on ice for 5 min, and then incubated at 16 °C for 30 min, 42 °C for 30 min, and 85 °C for 5 min, followed by a final incubation at 4 °C.

### 4.5. Real-Time Quantitative PCR (RT-qPCR) Analyses of miR-216a, miR-551b, miR-375, and miR-7

RT-qPCR assays of individual miRNAs were conducted in a total volume of 20 µL, comprising 1 µL of 20× TaqMan primer, 10 µL of 2× TaqMan^®^ Gene Expression Master Mix (Applied Biosystems; Catalog No. 4440047), 7.67 µL RNase-free water, and 1.33 µL diluted cDNA, according to the manufacturer’s instructions (User Manual No. 4364031 Rev. E, 01/2011, pages 16–18). The reactions were incubated at 95 °C for 10 min, followed by denaturation at 95 °C for 15 s and annealing at 60 °C for 60 s. The assays were performed in triplicate on a 7900 HT Fast Real-Time PCR System (Applied Biosystems). The expression level of U6 was used as a reference for normalization. The following miRNA probes (all Applied Biosystems) were used: *miR-551b* (cfa-miR-551b, dog, GCGACCCAUACUUGGUUUCAG), *miR-375* (cfa-miR-375, dog, UUUGUUCGUUCGGCUCGCGUGA), *miR-216a* (cfa-miR-216a, dog, UAAUCUCAGCUGGCAACUGUG), and *miR-7* (cfa-miR-7, dog, UGGAAGACUAGUGAUUUUGUUGU). Fluorescent signals were detected at the end of each cycle. The cycle threshold values were calculated using SDS 2.4 software (Applied Biosystems). The relative expression levels of the miRNAs were calculated using the 2^−ΔΔ*C*t^ method [24].

### 4.6. Pathology

Dogs were anesthetized by intramuscular injection of Alfaxan (Jurox, Hunter Valley, NSW, Australia) and Rompun (Bayer, Leverkusen, Germany), and then euthanized. Necropsy was performed on all animals, with macroscopic evaluation of the thoracic and abdominal cavities and tissues. After macroscopic examination, the excised pancreas was fixed in 10% neutral buffered formalin. The three lobes of the pancreas (head, body, and tail) were sectioned and dehydrated in graded ethanol, cleared in xylene using a Shandon Excelsior ES tissue processor (Thermo Fisher Scientific), and embedded in paraffin blocks using an EG1150H paraffin-embedding station (Leica Biosystems, Wetzlar, Germany). Histological examination of the pancreas was performed by an experienced veterinary pathologist (Woo-Chan Son, Asan Medical Center, Korea), and the lesions were semiquantitatively graded as minimal (+1), slight (+2), moderate (+3), marked (+4), or severe (+5).

### 4.7. Statistical Analysis

Values are expressed as the mean ± standard error of the mean. ANOVA was performed for multiple comparisons and Student’s *t*-test was used for comparisons between experimental groups by using GraphPad Prism 6 (GraphPad Software Inc., La Jolla, CA, USA). Fold changes in biomarker expression levels are expressed relative to those at the baseline or vehicle control. *p* < 0.05 was considered statistically significant.
